# Accelerating Treatment of Skeletal Class II Malocclusion using Fixed Twin Block Appliances

**DOI:** 10.5005/jp-journals-10005-1501

**Published:** 2018-04-01

**Authors:** Snigdha Pattanaik, Navya Puvvula, Noorjahan Mohammad

**Affiliations:** 1 Reader, Department of Orthodontics & Dentofacial Orthopedics Institute of Dental Sciences, Siksha O Anusandhan Deemed to be University, Bhubaneswar, Odisha, India; 2 Reader, Department of Orthodontics, Gitam Dental College & Hospital Visakhapatnam, Andhra Pradesh, India; 3 Reader, Department of Pedodontics, Mamata Dental College Khammam, Telangana, India

**Keywords:** Fixed orthodontics appliance, Fixed twin block appliance, Functional appliance, Growth modulation, Skeletal class II malocclusion.

## Abstract

**Background:**

Patients with class II malocclusion generally seek orthodontic treatment for esthetic concern. Various myo-functionl appliances can be used for the treatment of skeletal as well as the dental malocclusion in a growing patient. Among various functional appliances, twin block appliance is most commonly used due to better patient compliances. It redirects the mandibular growth to correct the maxillomandibular relationship, enhancing facial esthet ics. This article presents a modified design of the twin block appliance which is less bulky, more esthetic, can be used concurrently with fixed orthodontic appliance, and is easily accepted by uncooperative patients.

**Case summary:**

An 11-year-old-boy, who presented himself with a skeletal class II malocclusion, was treated with simultaneous use of fixed twin block along with fixed orthodontic appliance to correct both the skeletal and dental malocclusion. The twin block design was modified to have a better compatibility with the fixed orthodontic appliance.

**How to cite this article:** Pattanaik S, Puvvula N, Mohammad N. Accelerating Treatment of Skeletal Class II Malocclusion using Fixed Twin Block Appliances. Int J Clin Pediatr Dent 2018;11(2):146-150.

## CASE REPORT

**Figs 1A to D:**
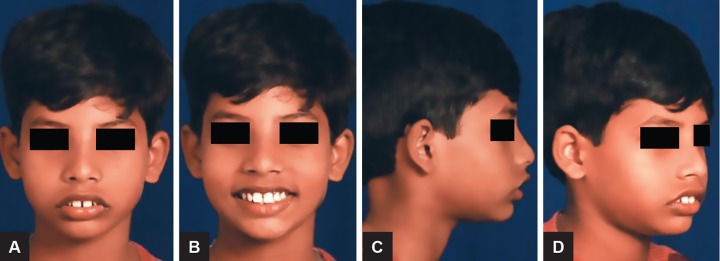
Pretreatment extraoral photographs

**Figs 2A to E:**
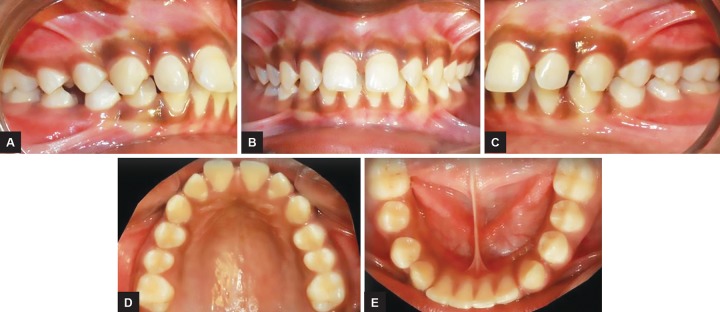
Pretreatment intraoral photograph

An 11-year-old male child presented reported to the Department of Orthodontics and Dentofacial Orthopedics with a chief complaint of forwardly placed upper front teeth. Clinical examination showed a convex facial profile due to mandibular retrusion and a mesoprosopic facial pattern. The interlabial gap was 8 mm with a short hypotonic upper lip and everted lower lip (Fig. 1). The intraoral examination revealed that the overjet was 8 mm and the overbite was excessive, but incomplete. The buccal segments were class II on both sides with class II canine relation. The maxillary arch showed spacing in the anterior region with mild rotation of both the second premolars. The mandibular arch also had spacing in the anterior region (Fig. 2).

**Fig. 3:**
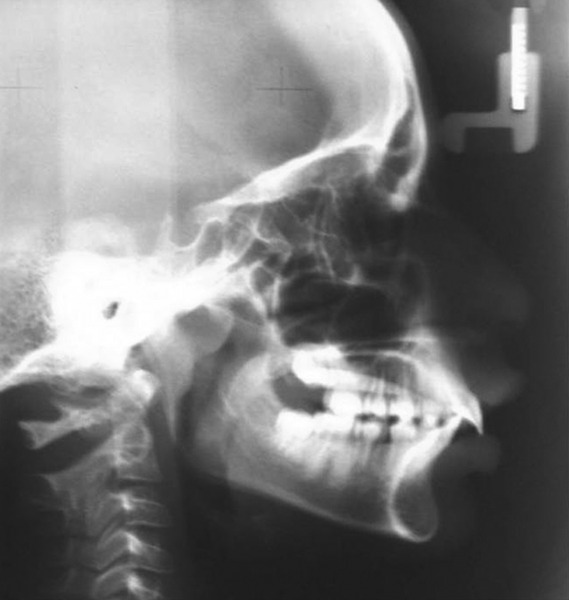
Pretreatment lateral cephalogram

**Fig. 4:**
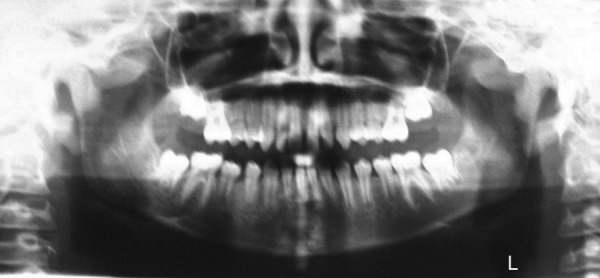
Pretreatment orthopantomogram

**Figs 5A and B:**
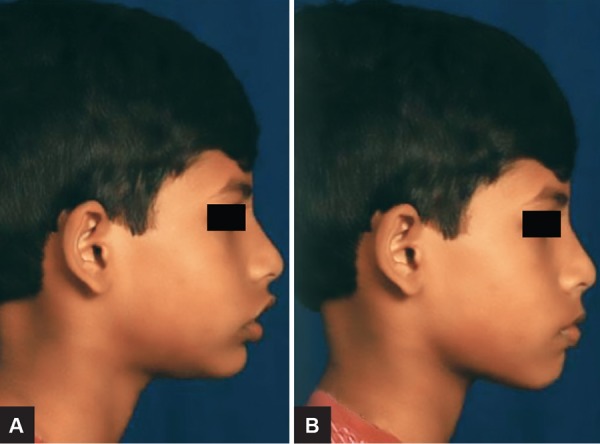
Profile view with VTO

**Fig. 6:**
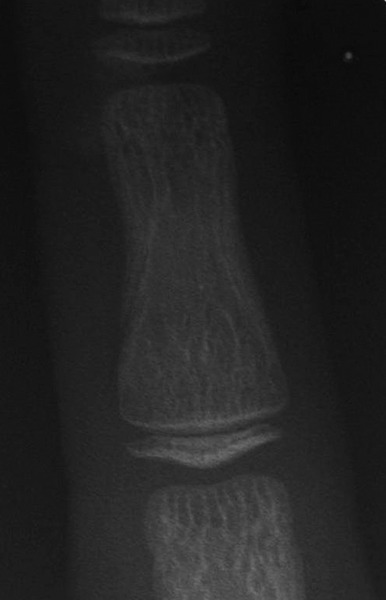
Mp3 radiograph

The cephalometric analysis confirmed a marked class II dental relationship with mandibular retrusion, average growth pattern, normal axial inclination of upper incisors, proclined lower incisors and acute nasolabial angle (Fig. 3). Orthopantomograph revealed that there was no underlying pathology or impacted teeth (Fig. 4). The visual treatment objective (VTO) of the patient was positive and suggested that the patient may be treated with growth modulation (Fig. 5). The mp3 radiograph and the cervical vertebra analysis suggested the acceleration of the curve of pubertal growth spurt and supported the growth modulation treatment plan (Fig. 6).

### Alternative Treatment Plans

- Correction of skeletal malocclusion first with a removable functional appliance followed by fixed orthodontic treatment to correct the dental malocclusion.

This two-step treatment would have increased the treatment duration. Patient cooperation is also essential for the completion of the treatment. So, this treatment plan was rejected.

- Fixed twin block appliance to correct the overjet along with the placement of fixed appliances to align both arches.

## FINAL TREATMENT PLAN AND TREATMENT RATIONAL

**Figs 7A to E:**
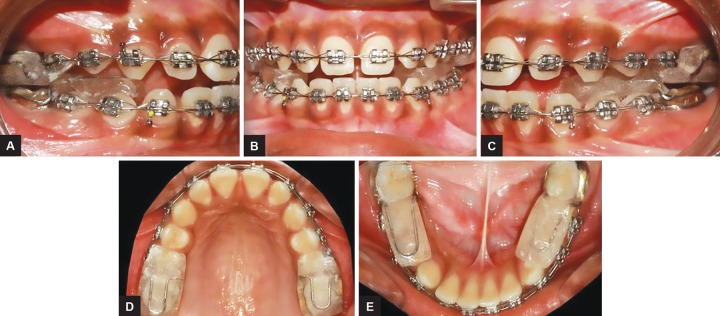
Modified twin block—intraoral view

The fixed twin block appliance along with fixed orthodontic appliance was chosen to eliminate patient compliance and to ensure full-time wear of the appliance (Fig. 7). The patient was advised to do lip exercise to achieve lip competency.

## TREATMENT PROGRESS

Preadjusted 0.022" MBT appliances were then bonded in both upper and lower arches.

**Fig. 8:**
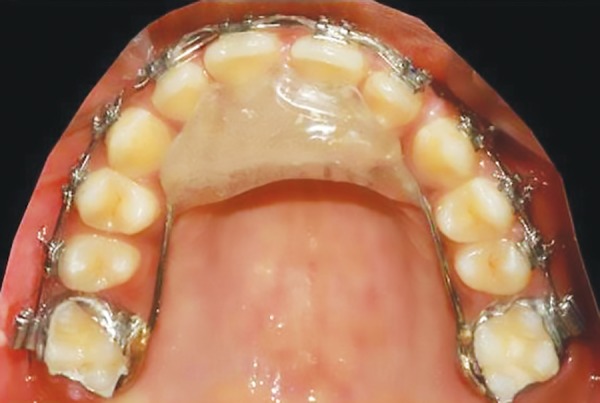
Retainer after active phase of twin block

The active twin block appliance phase lasted for 5 months. After the active phase of the appliances, the correction was maintained by placing an anterior inclined plane soldered to the upper first molars (Fig. 8).

**Figs 9A to C:**
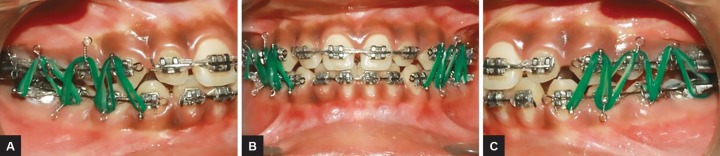
Class I elastics for settling of occlusion

Occlusal settling was done to obtain a good class I relation of the buccal segment (Fig. 9).

**Figs 10A and B:**
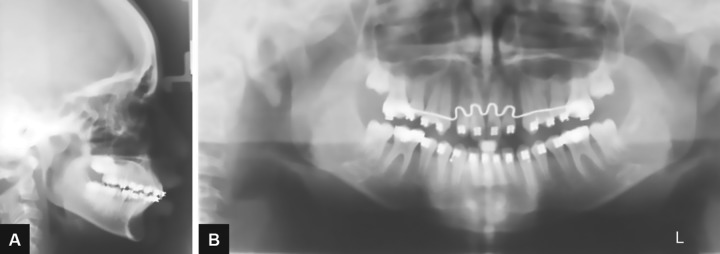
Posttreatment radiographs

Posttreatment radiographs show a marked improvement of the skeletal malocclusion with a decrease in the ANB angle. There was an increase in the effective length of the mandible, there by reducing the convexity of the profile. The improvement of the lip competency was also evident (Fig. 10).

### Advantages of the Appliance

This modified design of the twin block contains only the acrylic bite blocks, so it is less bulky and ensures better oral hygiene. The appliance can be used simultaneously with the fixed appliance, which shortens the treatment time, as it ensures full-time wear of the appliance. The presence of space between inclined planes and tooth surface permits tooth movement. It needs less patient compliance, as it is directly soldered to the molar bands.

## DISCUSSION

The twin block appliance was introduced in India in October 1989 by Dr William Clark. This is a removable orthopedic appliance to correct the skeletal malocclusion during the growth phase. It is actually made up of two acrylic bite blocks, one on the upper and the other on the lower arch, hence called “twin block.”^1^

Patients with skeletal class II malocclusions, who have growth potential, can be treated with appliances which modify or redirect growth. The principal appliances of choice are headgears and functional appliances. Functional appliances are the appliances of choice for treating class II malocclusions with a short and backwardly positioned mandible.^2^ Of the various functional appliances available, the twin block appliance has two major advantages of attributing to greater mandibular growth and less altered speech.^3^

In this patient, there was a requirement for the correction of dental as well as skeletal malocclusion. Fixed twin block appliance along with the fixed orthodontic appliance was hence the treatment of choice. Significant changes were seen within 2 to 3 months of use of twin blocks. The changes can be attributed to the altered muscle balance due to continuous wear of the appliance.

The twin block appliance consists of two acrylic bite blocks which are inclined at an angle of 70° to each other. The mechanism of action of the twin block depends on the functional displacement of mandible to a favorable position to correct the maxillomandibular discrepancy. The two piece design of the twin block enables the patient to use the appliance round the clock which improves orofacial function by bringing about both dental and skeletal changes.^4^

We have modified the original design of twin block by giving only the occlusal bite blocks, which rested on a stainless steel wire framework. The wire framework was made using a 0.9 mm stainless steel wire which was soldered to the molar bands in both upper and lower arches. The bite planes were made on the wire framework with self-cure clear acrylic. The bite blocks were inclined by 70° to each other.

The ideal timing for growth modulation for man-dibular deficiency is after the onset of pubertal growth spurt. A single-phase therapy where the orthopedic phase and orthodontic treatment are done at the same time is preferable, as studies have demonstrated that very early treatment involving two separate phases of therapy does not have any additional benefits.^5-7^

Slight overcorrection of the buccal segments (molars and canines) to a super class I position, which allows for a slight amount of relapse, is recommended when functional appliances are used.^8^ Correction of the skeletal malocclusion was maintained with an inclined bite plane incorporated in a Hawley’s appliance during the retention period. The amount of skeletal correction achieved is listed in Table 1.

**Fig. 11:**
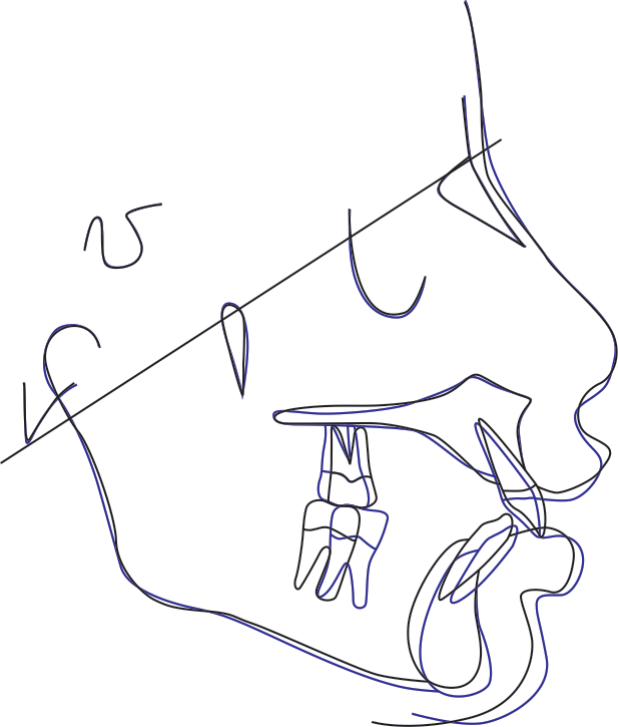
Superimposition of pre- and posttreatment radiographs

Superimposition of the lateral cephalograms taken before and after the functional phase therapy showed the following changes in the patient (Fig. 11):

- No skeletal changes were seen in the maxilla when the lateral cephalograms were superimposed on the Basion-Nasion line.
- The maxillary molar, however, showed a slight dis-talization effect.
- When superimposed on the internal structures of the mandible, a considerable increase was seen in the length of the body of mandible.
- The mandibular molar also showed a mesial movement which can be attributed to the growth of the mandible in the forward direction.
- Overall, superimposition at the cranial base showed:
  – Skeletal changes in the mandible with an increased mandibular body length.
  – Mesialization of mandibular molars.
  – Mild proclination of lower incisors as compared with the pretreatment cephalogram.
  – Improved soft tissue profile with a better prominence of the chin.

**Table 1:** **Table 1:** Comprehensive cephalometric evaluation

*Measurements*		*Normal values*		*Pretreatment*		*Posttreatment*	
SNA		80° ± 2°		84°		84°	
SNB		80° ± 2°		75°		79°	
ANB		2° ± 2°		9°		5°	
Co-Gn-				115 mm		120 mm	
Mandibular length							
Co-Point A				100 mm		100 mm	
Na I to Pog		Small, -8 to -6		–7 mm		–1 mm	
I to SN		102°		1,040		1,050	
I to PP		720		670		700	
T to MP (IMPA)		90		1,090		1,050	
T to NB		25°		390		370	
Interincisal angle		132°		1,140		1,160	
I to NA		4 mm		3 mm		2 mm	
T to Apog		2-4 mm		3 mm		2 mm	
T to NB		4 mm		8 mm		7 mm	

## CONCLUSION

The twin block appliance significantly increases mandibu-lar length as compared with the normative growth. They correct the underlying skeletal malocclusion and reduce the treatment duration of fixed orthodontic appliance. The modified design of the twin block described in this article can be used in uncooperative patients for better treatment results.
